# Machine learning-assisted analysis for agronomic dataset of 49 Balangu (*Lallemantia iberica* L.) ecotypes from different regions of Iran

**DOI:** 10.1038/s41598-022-23335-1

**Published:** 2022-11-10

**Authors:** Jalil Shafagh-Kolvanagh, Hassan Dehghanian, Adel Dabbagh Mohammadi-Nassab, Mohammad Moghaddam, Yaegoob Raei, Saeid Zehtab Salmasi, Peyvand Samimifar, Soheila Abdoli, Behnam Gholizadeh-Khajeh

**Affiliations:** 1 Department of Plant Ecophysiology, University of Tabriz, Tabriz, Iran; 2 Department of Plant Biotechnology and Breeding, University of Tabriz, Tabriz, Iran

**Keywords:** Evolution, Plant sciences, Climate sciences

## Abstract

The Balangu (*Lallemantia iberica*) species have a high gastronomical impact in the Middle East and Balkan region. It is widely used in the local food industry, such as confectionery, edible oil, and protein food. In this study, 49 ecotypes were collected from different regions of Iran. 37 agronomic traits were measured during the growing season and at harvest time. To find the correlation between the grain yield per unit area, grain yield per single plant (GYSP), oil percent (OP), and protein percent (PP) with other measured traits, which these were utilized as the labels of different machine learning (ML) procedures including Linear Regression (LR), Support Vector Regression (SVR), Random Forest Regression (RFR), and Gradient Boosting Decision Tree Regression (GBDTR). It was observed that there is a linear relationship between the measured agronomic traits and the considered labels. So, the LR, RFR, and GBDTR models showed the lowest mean absolute error, mean square error, and root mean square error than SVR models and good prediction ability of the test data. Although, the RFR and GBDTR have naturally lower bias than other methods in this study, but the GBDTR scheme is preferred because of the over-fitting shortcoming of the RFR technique. The GBDTR method showed better results rather than the other ML regression methods according to the RMSE 3.302, 0.040, 0.028, and 0.060 for GYUA, GYSP, OP, and PP, respectively.

## Introduction

The Balangu (*Lallemantia Iberica*) species are cultivated in the different regions of Iran with historical roots in the gastronomic culture^1–3^. This plant also has been used for medical applications^4^. In a new development, *Lallemantia* species are being used to synthesize gold and silver nanoparticles without hazardous materials^5^. Recently, effective symbiosis under drought stress has been reported for *Lallemantia iberica*^6^. Clearly, it could be used in soils with high salt concentration and improve the characteristics of the soil after harvest. Similarly, it has been found that one of the *Lallemantia* species could be used in wastewater treatment effectively and environmentally, especially in the semi-arid regions^7^. Accordingly, this plant family has great adaptability to various environmental conditions and could be used as ecosystem services to improve the soil health and quality of the water resources of the cultivated region.

The *Lallemantia iberica* contains protein and oil in its structure which is the reason for the gastronomical uses of this plant. Unfortunately, insufficient data are available about the agronomic characteristics of these species and the effect of ecotype on extracted compounds such as protein and oil. Therefore, studies about the agronomic parameters of the *Lallemantia iberica* are required.

In the state of the art, machine learning (ML) has been used in the data analysis to predict yield responsiveness to nitrogen fertilization in maize^8^ and predicting grain arsenic concentration in rice under deficit irrigation system and use of organic amendments^9^, and crop yield forecasting^10,11^. However, to our best knowledge, there is no such investigation to study the relationship of the agronomic characteristics with the grain yield. The ML procedures could be used to solve classification, clustering, and regression problems.^10^ The regression is one of the supervised applications of the ML methods that could provide a reliable result in the prediction of a complex dataset^11^. The regression application can use various algorithms such as linear regression with a diversity of modifications, such as random forest regression (RFR), support vector regression (SVR), and gradient boosting decision tree regression (GBDTR)^12–14^.

The present work provides a four-year dataset for the agronomic properties of *Lallamentia iberica* and the metrics such as morph-physiological traits of 49 different ecotypes from various regions of Iran with the diversity of extracted materials percent’s such as protein and oil. Machine Learning is used for data analysis. The prediction for the grain yield performance was the main subject of this analysis. In this respect, linear, support vector machine regression, random forest regression, and gradient boosting decision tree have been utilized.

## Results and discussion

The measured traits of the *Lallamentia iberica* ecotypes and their descriptive data, including the mean, standard deviation, minimum, maximum, and quartiles, averaged over four years, are shown in Tables 1 and 2. Based on the descriptive data, the mean of 49 ecotypes for oil and protein content was 38.59% and 21.20%, respectively, indicating that *Lallamentia iberica* is rich in oil and protein. Likewise, the grain yield per unit area could reach the maximum value of 169 g/m^2^ (1690 kg/ha) in this experiment.

**Table 1:** The measured traits of the *Lallemantia iberica* ecotypes with corresponding abbreviations, and units of measurement.

Trait	Abbreviation	Unit
Biomass per unit area	BYPUA	g m^−2^
Straw yield per unit area	SYUA	g m^−2^
Grain yield per unit area	GYUA	g m^−2^
Biomass yield per plant	BYP	g
Straw yield per plant	SYP	g
Grain yield per plant	GYP	g
Number of seeds per plant	NSP	–
Plant height	PH	cm
Stem diameter	SD	mm
Number of nodes in the main stem	NNMS	–
Number of leaves in the main stem	NLMS	–
Number of fertile sub-branches	NFSB	–
Fertile branch length	FBL	cm
Number of nodes in the fertile sub-branch	NNFSB	–
Number of leaves per fertile branch	NLFB	–
Number of capsules in the main stem	NCMS	–
Number of capsules in each sub-branch	NCSB	–
Number of flower cycles in the main stem	NFCMS	–
Number of flower cycles in the sub-branch	NFCSB	–
Number of capsules per cycle in the main stem	NCCMS	–
Number of capsules in each cycle in the sub-branch	NCCSB	–
Number of seeds in the main stem	NSMS	–
Number of seeds per branch	NSB	–
Number of seeds per the main cycle	NSMC	–
Number of seeds per sub-cycle	NSSC	–
1000-grain weight per unit area	TGWUA	–
Harvest index per unit area	HIUA	–
Oil percentage	OP	%
Oil yield per unit area	OYUA	–
Protein percentage	PP	%
Protein yield per unit area	PYUA	–
Oil extraction index per unit area	OEIUA	–
Protein extraction index per unit area	PEIUA	–
Chlorophyll index	CHI	–
Leaf area index	LAI	–
Oil yield per plant	OYP	–
Protein yield per plant	PYP	–

**Table 2:** The descriptive data of the *Lallemantia iberica* ecotypes, evaluated during the 2014–2017 growing seasons.

Trait^1^	Mean	Standard deviation	Minimum	Quartile			Maximum
				25%	50%	75%	
BYUA	274.42	80.80	120.00	215.93	261.10	318.35	565.32
SYUA	187.36	62.45	58.96	143.51	175.64	218.34	446.42
GYUA	87.07	24.16	15.32	70.51	83.96	102.86	169.30
BYP	2.03	0.65	0.65	1.58	1.95	2.42	4.55
SYP	1.35	0.45	0.42	1.03	1.30	1.59	3.40
GYP	0.68	0.22	0.12	0.53	0.67	0.82	1.55
NSP	122.09	40.71	25.20	93.05	116.15	146.23	277.71
PH	30.30	4.89	19.40	26.76	30.00	33.52	43.10
SD	2.01	0.42	1.11	1.72	1.96	2.22	4.75
NNMS	13.31	1.54	8.40	12.30	13.50	14.40	16.90
NLMS	26.24	2.98	16.80	24.40	26.60	28.20	45.00
NFSB	2.40	1.14	0.00	1.60	2.30	3.00	7.40
FBL	10.51	3.42	0.00	8.12	10.34	12.74	22.17
NNFSB	5.23	1.10	0.00	4.50	5.30	6.00	8.37
NLFB	10.08	2.08	0.00	8.80	10.01	11.61	16.75
NCMS	36.61	6.79	13.70	32.38	36.75	40.90	59.40
NCSB	8.72	3.36	0.00	6.42	8.50	10.87	24.04
NFCMS	8.21	1.16	4.20	7.70	8.21	8.90	11.89
NFCSB	4.00	1.01	0.00	3.49	4.00	4.66	6.74
NCCMS	5.36	0.52	3.68	5.09	5.36	5.61	7.26
NCCSB	2.78	0.68	0.00	2.47	2.78	3.12	4.92
NSMS	2.86	0.60	1.02	2.44	2.81	3.20	5.40
NSPMS	102.00	26.92	38.67	87.70	102.00	111.38	227.22
NSB	21.22	9.12	0.00	15.46	21.22	24.08	54.19
NSMC	14.66	2.92	7.16	12.99	14.66	15.67	29.90
NSSC	7.61	2.41	0.00	6.25	7.61	8.53	17.27
TGWUA	4.98	0.34	3.80	4.80	5.00	5.20	5.90
HIUA	32.25	5.38	10.80	29.20	32.29	35.10	51.52
OP	38.59	1.72	32.81	37.67	38.59	39.41	45.77
OYUA	31.26	7.11	13.44	26.93	31.26	34.44	59.93
PP	21.20	2.47	15.64	19.82	21.20	21.76	34.38
PYUA	17.35	4.33	7.36	14.72	17.35	18.90	32.68
OEIUA	38.59	1.72	32.81	37.67	38.59	39.41	45.77
PEIUA	21.20	2.47	15.64	19.82	21.20	21.76	34.38
CHI	20.02	4.92	9.40	16.98	20.02	21.13	39.35
LAI	1.27	0.43	0.38	0.98	1.27	1.40	3.06
OYP	0.22	0.04	0.07	0.22	0.22	0.22	0.51
PYP	0.12	0.02	0.04	0.12	0.12	0.12	0.29

^1^The abbreviations are based on Table 1; The units for the measured traits can be found in Table 1.

In this study, four labels, grain yield per unit area (GYUA), grain yield per plant (GYP), oil percent (OP), and protein percent (PP) of the *Lallamentia iberica* ecotypes were separately used for the sake of prediction purpose via other agronomic traits based on regression. The results and discussion for each label are briefly stated here.

### GYUA

**Figure 1:**
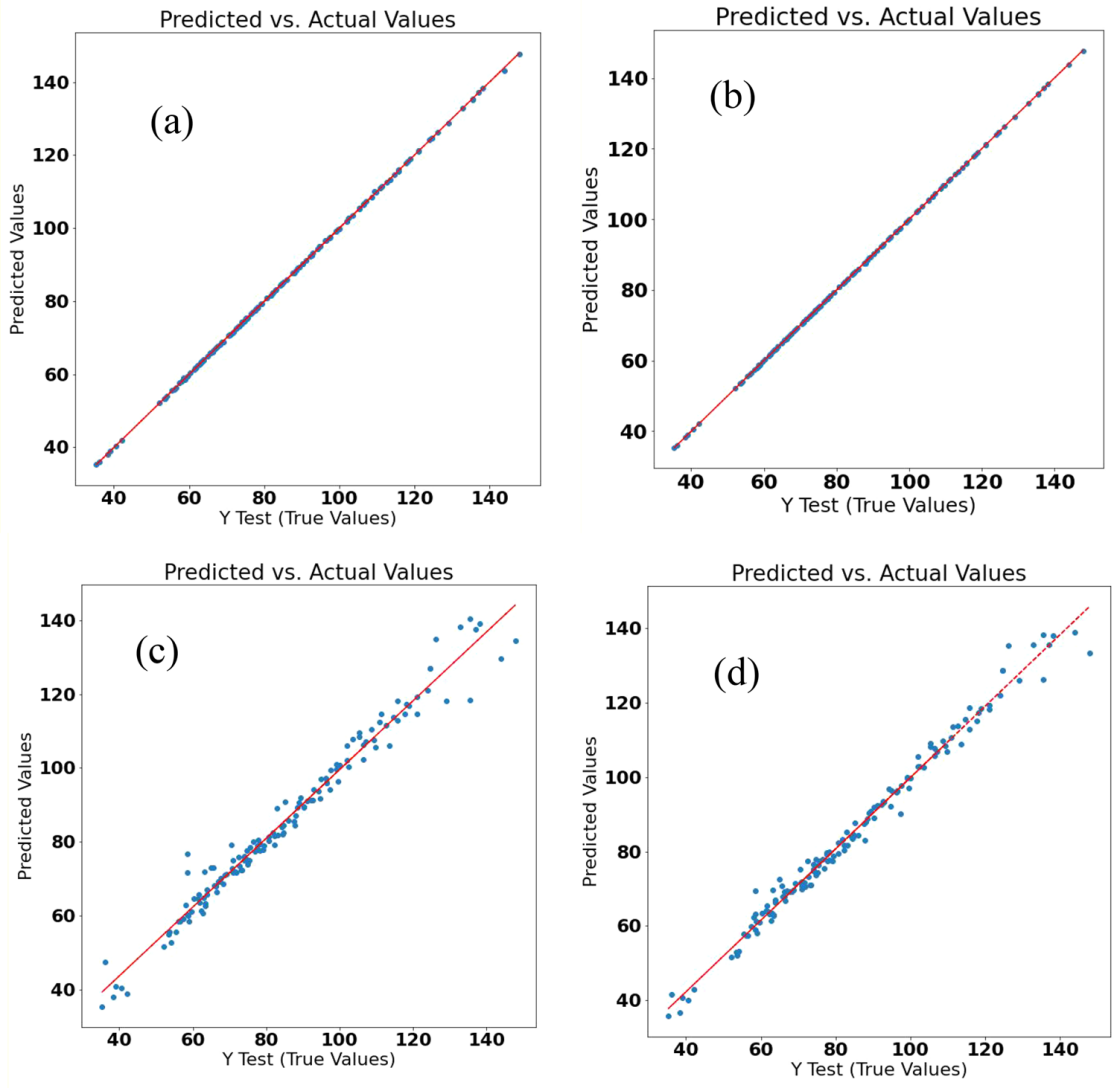
(**a**) Linear (**b**) Support vector, (**c**) Random forest, and (**d**) gradient boosting regressions of the grain yield per unit area of the *Lallemantia iberica* ecotypes based on machine learning regression.

The prediction results of the GYUA through the ML-based regression methods are depicted in Table 3 and Fig. 1. According to Table 3, the LR, SVR Linear Kernel, RFR, and GBDTR methods had much lower mean absolute error (MAE), mean square error (MSE), and root means square error (RMSE) than SVR Gaussian Kernel, SVR Polynomial Kernel, and SVR Sigmoid Kernel. Figure 1 demonstrates the predicted values versus the experimentally evaluated values of the GYUA label for the LR, SVR Linear Kernel, RFR, and GBDTR methods had good prediction results. The main goal of this sequence of the ML methods was to eliminate possible overfitting and the potential bias in the learning process linear regression and increase the accuracy of the analytical information. Although the LR and SVR Linear Kernel methods showed a slightly better prediction ability than the other two methods, their higher biases may affect the objectivity of the results in this case. The Random forest and gradient boosting decision tree regression are used to import the random states to avoid the created bias of the linear ML regression algorithms. While the RFR may better handle the bias, under-fitting might be a problem^15^. On the other hand, the GBDTR as a hybrid method avoids the issues of the previous methods. The RFR and GBDTR methods are used to import the random states to avoid the created bias of the linear ML regression algorithms^16^.

**Table 3:** The mean absolute error (MAE), mean square error (MSE), root mean square error (RMSE), and area under curve (AUC) of receiver operating characteristics (ROC) of the applied machine learning (ML) regression methods in predicting the grain yield per unit area of the *Lallemantia iberica* ecotypes with the training of other traits, using the K-fold cross-validation as the data splitting method.

ML method	AUC-ROC	MAE	MSE	RMSE
Linear regression	0.9969	0.176	0.760	0.871
Support vector regression (SVR); linear kernel	0.9979	0.120	0.681	0.825
SVR; Gaussian kernel	0.8157	11.981	250.224	15.818
SVR; polynomial kernel	0.8404	11.068	243.275	15.597
SVR; sigmoid kernel	0.2229	22.008	829.985	28.809
Random forest regression	0.9540	2.537	15.648	3.955
Gradient boosting decision tree regression	0.9695	2.097	10.907	3.302

**Figure 2:**
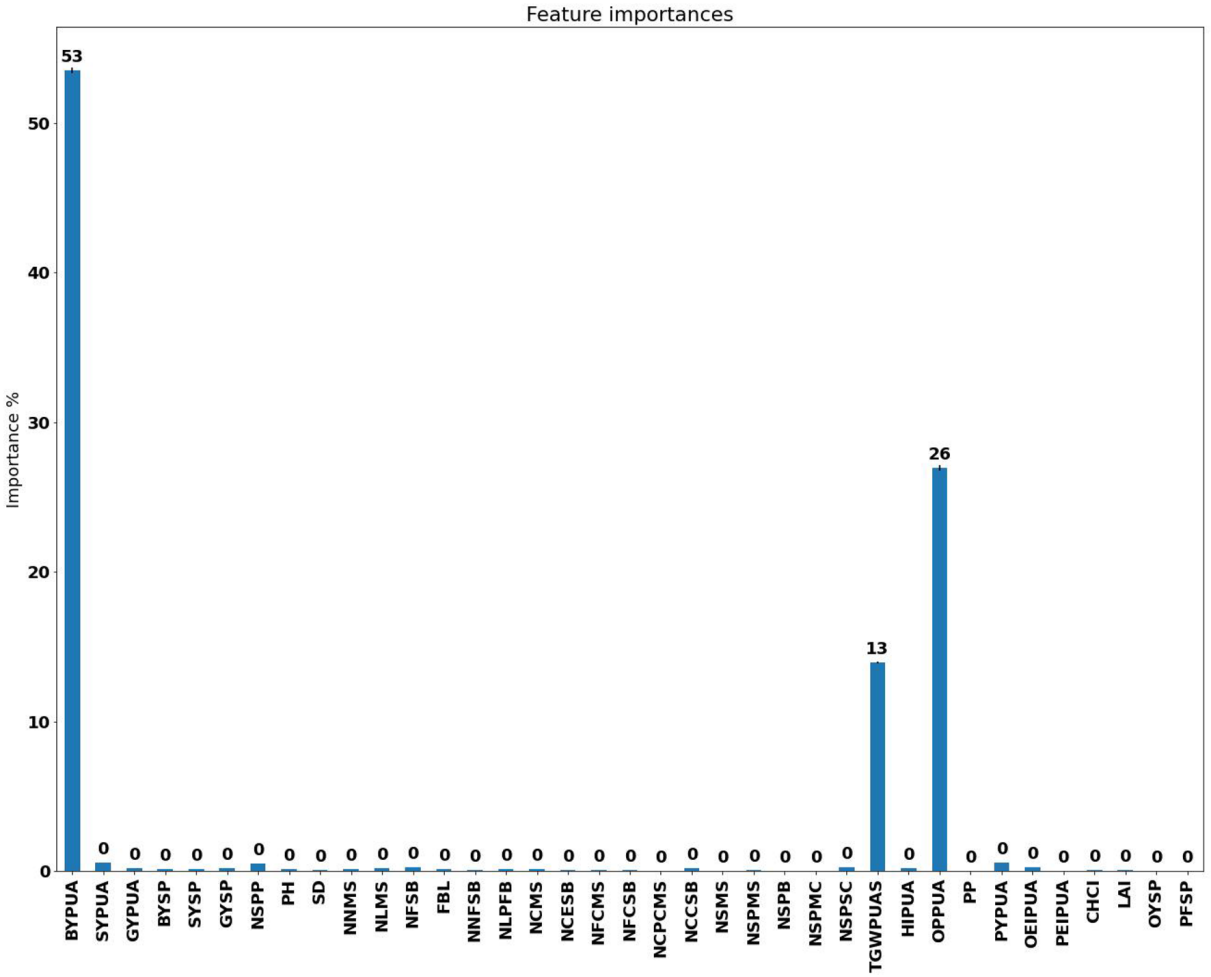
The importance of the traits in the random forest machine learning regression process for the prediction of the grain yield per unit area of the *Lallemantia iberica* ecotypes.

There was a linear relationship between the indicator features and the label. In this respect, the effect of different features was evaluated in the ML process. For example, BYUA, OYUA, and HIUA were the most important features for predicting GYUA in RFR, respectively (Fig. 2). Hence, BYUA, OYUA, and HIUA can be regarded as good predictor indicators of GYUA. Similar results were reported for sesame (*Sesamum indicum* L.) in another study^17^.

### GYSP

**Figure 3:**
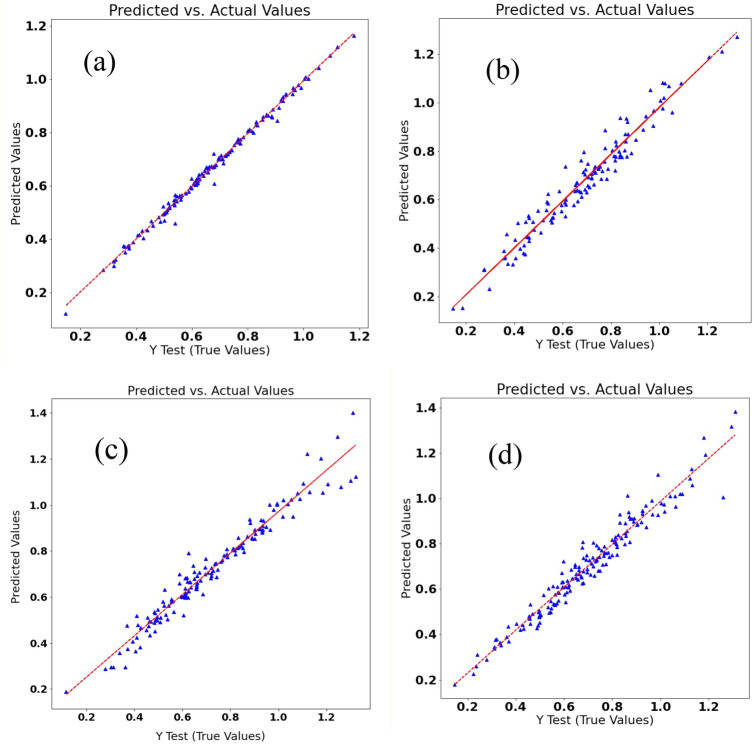
(**a**) Linear (**b**) Support vector, (**c**) Random forest, and (**d**) gradient boosting regressions of the grain yield per plant of the *Lallemantia iberica* ecotypes.

The grain yield of a single plant is an important trait in crop yield evaluation and genetic assessments^18,19^. The results of the assessment of different ML methods in the prediction of GYSP by MAE, MSE, and RMSE are shown in Table 4. Although LR had slightly better performance than RFR and GBDTR in the prediction of GYSP (Table 4; Fig. 3), the bias in this method makes it partially unreliable^20^, as previously mentioned. Despite the fact that the RFR and GBDTR methods demonstrated higher error rather than LR and SVR Linear Kernel^21^ and the generated random states in the learning process could disturb the prediction, the results are more reliable due to the reduced bias.

**Table 4:** The mean absolute error (MAE), mean square error (MSE), root mean square error (RMSE), and area under curve (AUC) of receiver operating characteristics (ROC) of the applied machine learning (ML) regression methods in predicting the grain yield per plant of the *Lallemantia iberica* ecotypes with the training of other traits, using the K-fold cross-validation as the data splitting method.

ML method	AUC-ROC	MAE	MSE	RMSE
Linear Regression	0.9667	0.010	0.001	0.018
Support vector regression (SVR); linear kernel	0.9302	0.038	0.002	0.048
SVR; Gaussian kernel	0.8775	0.069	0.007	0.086
SVR; polynomial kernel	0.8862	0.070	0.007	0.086
SVR; sigmoid kernel	0.3099	4.346	33.50	5.788
Random forest regression	0.9372	0.031	0.002	0.044
Gradient boosting decision tree regression	0.9503	0.030	0.002	0.040

**Figure 4:**
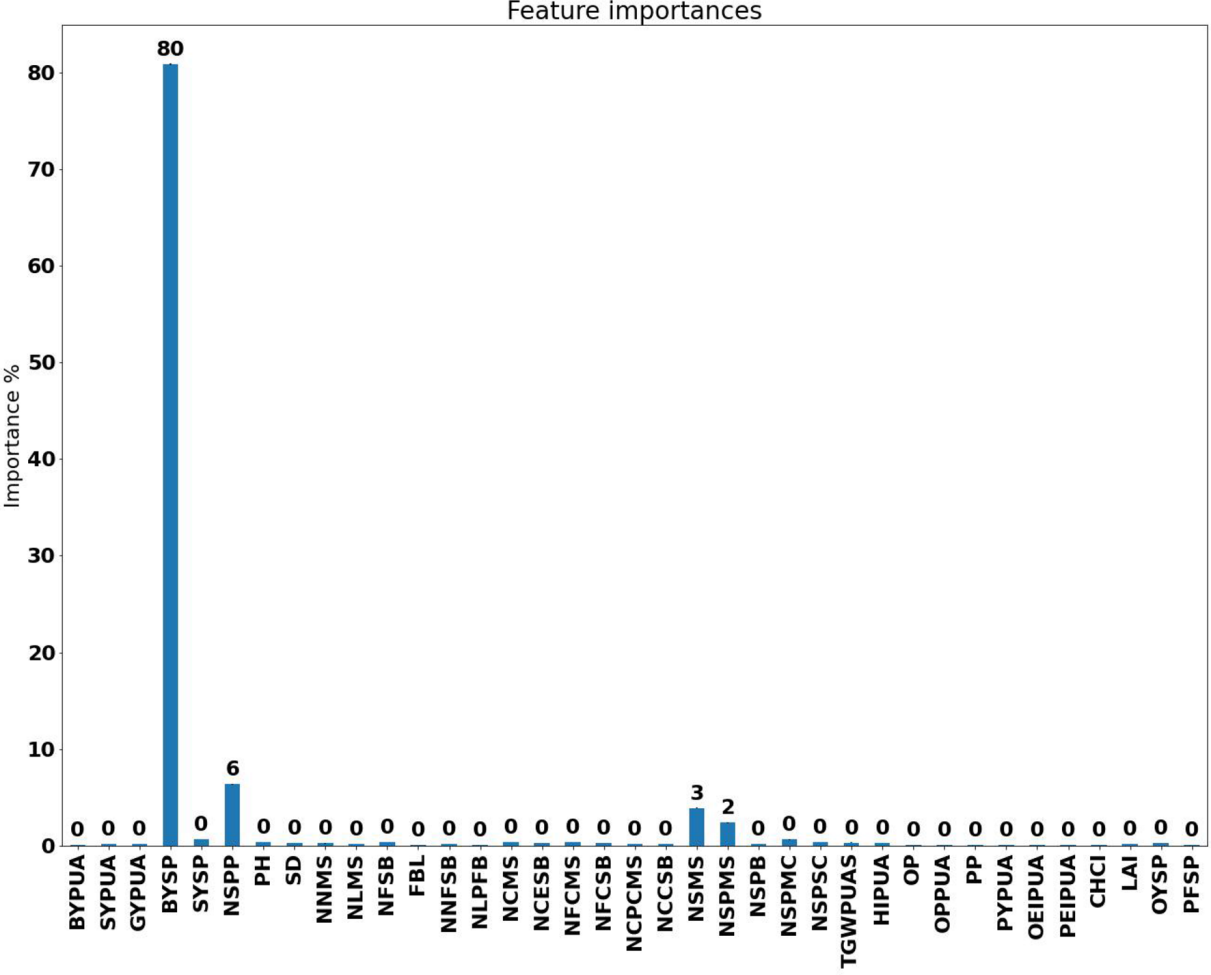
The importance of the traits in the random forest machine learning regression process for the prediction of the grain yield per plant of the *Lallemantia iberica* ecotypes.

As an example of the importance of the indicator in the prediction of GYSP, the results for the RFR method were depicted in Fig. 4. The features BYSP, NSPP, and NSMS were the most effective features on GYSP in the RFR method, respectively. Besides, NSPSC had a small impact on this label.

### OP

**Figure 5:**
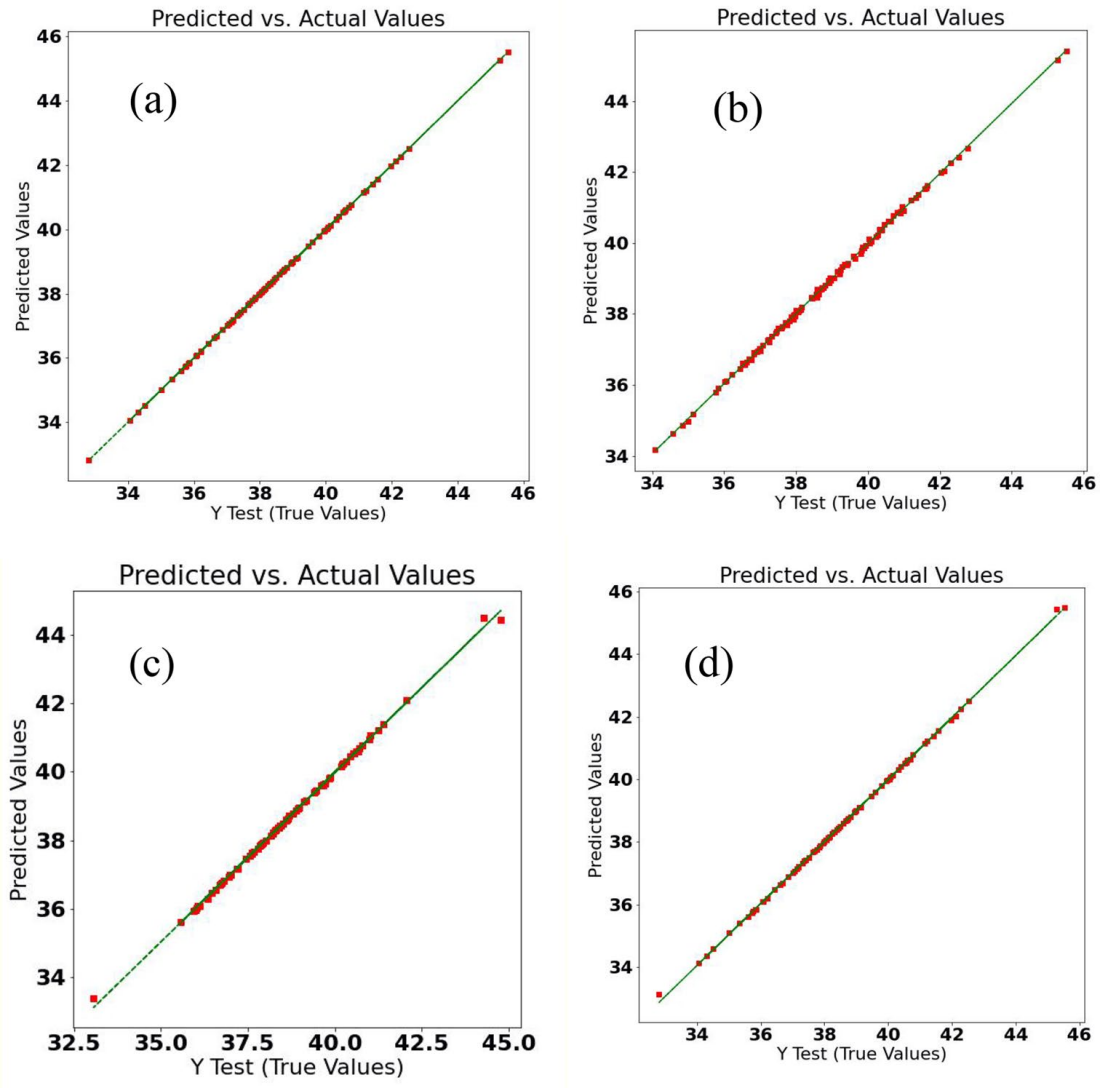
(**a**) Linear (**b**) Support vector, (**c**) Random forest, and (**d**) gradient boosting regressions of the oil percentage of the *Lallemantia iberica* ecotypes.

Oil percentage is an essential trait with respect to the gastronomic point of view^22^. *Lallamentia iberica’*s oil is healthy in food production and other uses in the cultivated region. The efficiency of different ML methods in the prediction of the OP was compared in Table 5. The GBDTR method showed the best performance in predicting OP followed by RFR and SVR Linear Kernel. Figure 5 shows the predicted values versus the tested values of the OP label for the LR, SVR Linear Kernel, RFR, and GBDTR methods. All four methods were acceptable in predicting OP. However, the different conditions should be considered to achieve an appropriate approach with the corresponding practical state. For example, if OP needs to be predicted for a specific ecotype, the gradient boosting decision tree would be a good choice. At the same time, in the particular condition with the features, the SVR could be used efficiently.

**Table 5:** The mean absolute error (MAE), mean square error (MSE), root mean square error (RMSE), and area under curve (AUC) of receiver operating characteristics (ROC) of the applied machine learning (ML) regression methods in predicting the oil percentage of the *Lallemantia iberica* ecotypes with the training of other traits, using the K-fold cross-validation as the data splitting method.

ML method	AUC-ROC	MAE	MSE	RMSE
Linear regression	0.9998	1.145	1.851	1.360
Support vector regression (SVR); linear kernel	0.9987	0.047	0.003	0.056
SVR; Gaussian kernel	0.5987	1.178	2.875	1.696
SVR; polynomial kernel	0.6076	1.169	2.847	1.687
SVR; sigmoid kernel	0.4847	4.685	39.60	6.292
Random forest regression	0.9970	0.023	0.002	0.054
Gradient boosting decision tree regression	0.9963	0.014	0.0007	0.028

### PP

**Figure 6:**
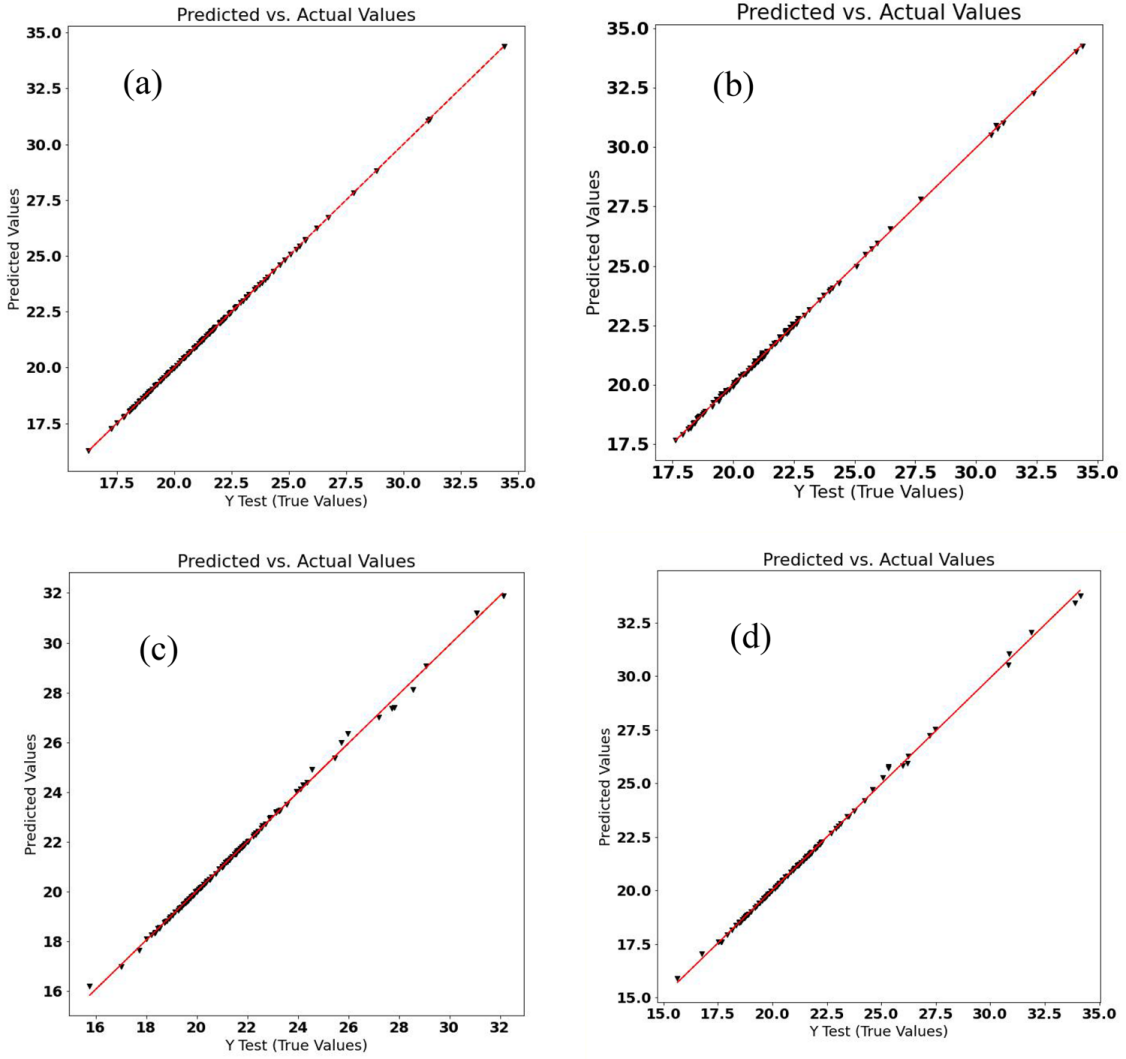
(**a**) Linear (**b**) Support vector, (**c**) Random forest, and (**d**) gradient boosting regressions of the protein percentage of the *Lallemantia iberica* ecotypes.

*Lallamentia iberica* is a protein reach plant that contains 21% protein on average with a maximum of 34%^23,24^. In this respect, the protein content of this plant was another subject of this work. The results of the prediction of PP by other agronomic characteristics for the LR, SVR Linear Kernel, RFR, and GBDTR methods are demonstrated in Fig. 6. A very high efficient prediction has been achieved with ML regression methods. The results were similar to previous labels. A linear relationship was observed between PP and the measured characteristics of this species. The estimated MAE, MSE, and RMSE in the prediction of PP are given in Table 6. The GBDTR method was the most effective procedure in predicting PP followed by RFR and SVR Linear Kernel methods. GBDTR trains the data with random decision trees and also performs a linear regression based on these data. So, the predicted PP by this method would be more reliable than the other methods.

**Table 6:** The mean absolute error (MAE), mean square error (MSE), root mean square error (RMSE), and area under curve (AUC) of receiver operating characteristics (ROC) of the applied machine learning (ML) regression methods in predicting the protein percentage of the *Lallemantia iberica* ecotypes with the training of other traits, using the K-fold cross-validation as the data splitting method.

ML method	AUC-ROC	MAE	MSE	RMSE
Linear regression	0.9971	1.020	1.522	1.233
Support vector regression (SVR); linear kernel	0.9909	0.045	0.004	0.064
SVR; Gaussian kernel	0.6624	1.871	9.539	3.088
SVR; polynomial kernel	0.6982	1.855	9.318	3.052
SVR; sigmoid kernel	0.5528	4.339	31.841	5.643
Random forest regression	0.9952	0.037	0.020	0.143
Gradient boosting decision tree regression	0.9983	0.022	0.003	0.060

## Conclusions

The descriptive data showed that *Lallamentia iberica* is rich in oil and protein with the four-year average of 38.59% and 21.20%, respectively. Furthermore, it was shown that the grain yield per unit area could reach up to 1690 kg/ha in this experiment. The ML regression methods showed that there was a linear relationship between the indicator variables and GYUA, GYP, OP, and PP. ML Linear Regression, SVR Linear Kernel, RFR, and GBDTR had generally lower MAE, MSE, and RMSE than other SVR method and showed good fitting to the data set. Although both RFR and GBDTR have inherently lower bias than other utilized methods, the GBDTR method is a better choice since over-fitting is regarded as a disadvantage for the ML RFR.

## Materials and methods

**Figure 7:**
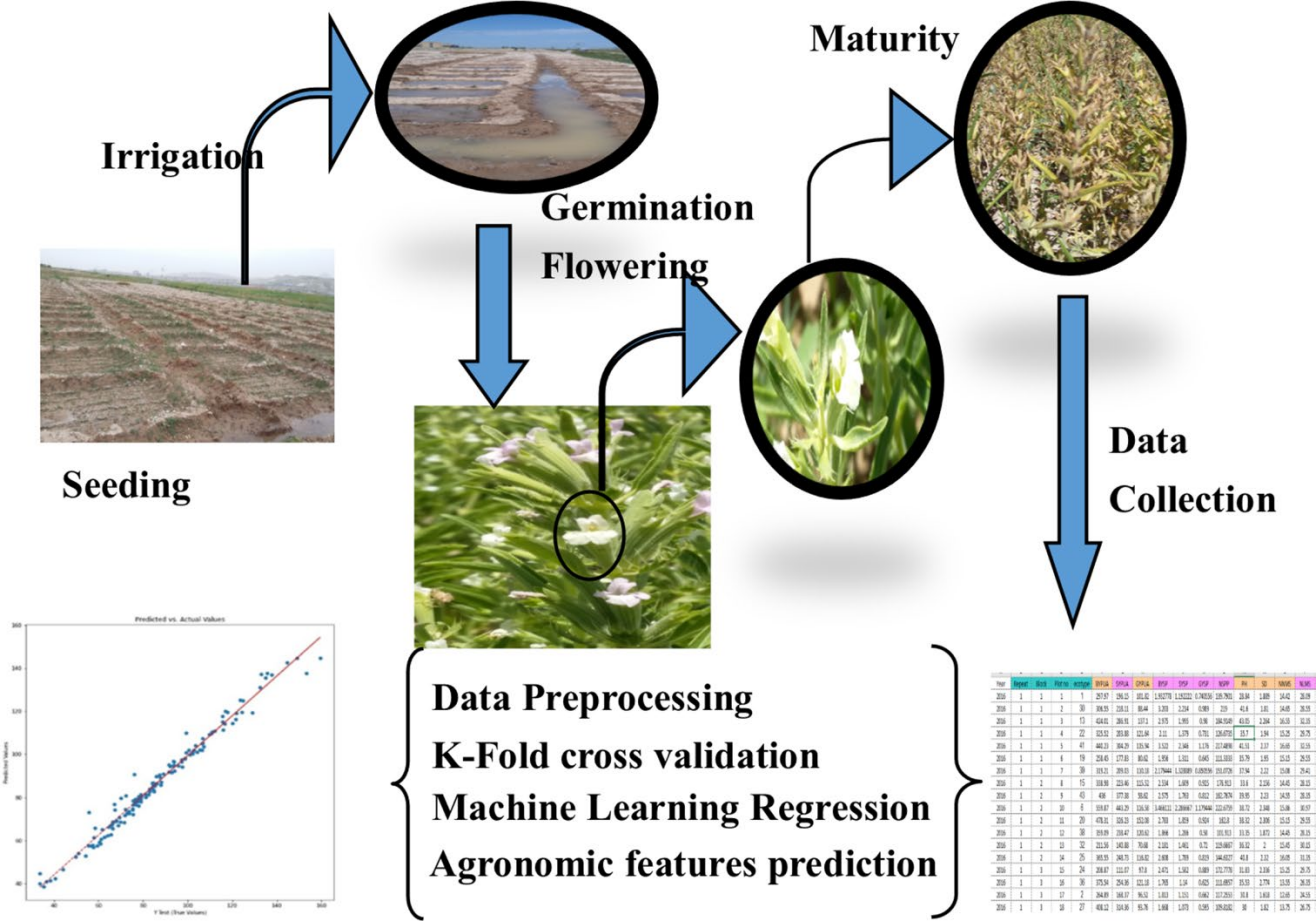
The schematic of the methodology has been implemented to study Balangu’s agronomic properties.

During years 2014–2017 an agronomic study has been implemented to evaluate the Balngu’s different agronomic properties. A machine learning approach is used to assess the evaluated agronomic properties. The whole process has been illustrated in Fig. 7. The detail of the measurement, data collection and data processing have been given in the next sections.

### Characteristics of the experimental site

This research was carried out in the research station of the Faculty of Agriculture, University of Tabriz, Tabriz, Iran. The experimental site was located at the longitude of 46° 17′, latitude of 38° 05′, and altitude of 1360 m. The annual rainfall was about 285 mm, averaged over four years. The average temperature was recorded about at 10 °C, with *T*_max_ = 16.6 °C and *T*_min_ = 4.2 °C. The physical properties of soil are given in Table 7. The soil type was silty loam with the pH of 7.75.

**Table 7:** The properties of soil in the experimental site.

Absorbable potassium (ppm)	Absorbable phosphorous (ppm)	Nitrogen (%)	Organic matter (%)	Soil type	Sand (%)	Silt (%)	Clay (%)	Acidity	Conductance (µS cm^−1^)
304	61	0.08	0.76	Sandy loam	65	20	15	7.75	475

### Plant material and experimental design

In the present investigation, 49 ecotypes were evaluated in four years (2014–2017), using the 7 × 7 triple design. The codes and locality of the ecotypes are listed in Table 8. Permission to collect the seeds of ecotypes was obtained from the farmers of each region before sampling. This study complies with relevant institutional, national, and international guidelines and legislation.

**Table 8:** Code and locality of *Lallemantia iberica* ecotypes were evaluated in this study.

Ecotype locality	Ecotype number	Ecotype locality	Ecotype code
Kalvanagh 15	E_26_	Kalvanagh 1	E_1_
Param 1	E_27_	Kalvanagh 2	E_2_
Zaranagh	E_28_	Kalvanagh 3	E_3_
Varzeghan 1	E_29_	Kalvanagh 4	E_4_
Ahar 1	E_30_	Ahar	E_5_
Tazeh-kand	E_31_	Kalvanagh 5	E_6_
Malekan	E_32_	Kalvanagh 6	E_7_
Mashhad	E_33_	Kalvanagh 7	E_8_
Varzeghan 2	E_34_	Sarab	E_9_
Param 2	E_35_	Kalvanagh 8	E_10_
Peygham Kaleyber	E_36_	Kalvanagh 9	E_11_
Alvar Bostan Abad	E_37_	Tabriz 2	E_12_
Dehlan Hashtrood	E_38_	Tabriz 5	E_13_
Jolfa Komar-Sofla	E_39_	Tabriz 3	E_14_
Bijar Gondak	E_40_	Tabriz 1	E_15_
Urmia Serow Border	E_41_	Tabriz 7	E_16_
Marand Arlan	E_42_	Tabriz 6	E_17_
Khalkhal Majara	E_43_	Tabriz 8	E_18_
Lilab Marand	E_44_	Kalvanagh 10	E_19_
Kharvana	E_45_	Kalvanagh 11	E_20_
Kurdistan 2	E_46_	Kalvanagh 12	E_21_
Takab	E_47_	Kalvanagh 13	E_22_
Zanjan	E_48_	Tabriz 4	E_23_
Nasarlu and Darvish Baghghal villages	E_49_	Kalvanagh 14	E_24_
	Tazeh-kand	E_25_	

Each plot consisted of five rows of 1.5 m in length, with the between-row distance of 20 cm and within-row spacing of 1 cm. The planting density was 500 seeds per m^2^. The planting date each year was the 4th of May. Standard cultural practices, such as soil fertilization, irrigation, and weed control were performed during the growing season. The harvesting date in each year was the 26th of July. The harvested area was 0.5 m^2^ of the plot center. During the growing season and at the harvest, 37 traits were measured in each plot.

To estimate leaf area index (LAI) the green leaf area per unit area of ground surface was determined^25^. Also, leaf chlorophyll index at the full-flowering stage using three random plants in each plot. In each plant, the chlorophyll index was measured from three parts (bottom, middle, top), using CCM-200 Plus (Opti-Sciences Inc., NH, USA).

### Data analysis

In this study, different ML regression methods were considered to predict the four labels, namely GYUA, GYP, OP, and PP by other measured traits. The ML regression methods included ML Regression, SVR Linear Kernel, SVR Gaussian Kernel, SVR Polynomial Kernel, SVR Sigmoid Kernel, RFR, and GBDTR were used to analyze the four-years data set^26–31,32,33^. The K-fold cross-validation was used to split the training and the test data sets. Furthermore, MAE, MSE, and RMSE were utilized to compare the efficiency of the ML regression methods.

### Machine learning regression

#### Linear regression

Supervised learning is the base of the linear regression in the machine learning method with a target prediction of the independent variable^34^.

1 $$y = \theta_{1} + \theta_{2} x$$

In this equation, *x* is input training data (univariate—one input variable (parameter)), and *y* is labeled to data (supervised learning). In the training process, model the best line to predict the value of *y* for a given value of *x* is fitted. The process would provide the best deals for the *θ*_*1*_ (intercept) and *θ*_*2*_ (coefficient of *x*) after fitting. Afterward, the coefficient would predict the *y* value with a given *x* data in the test process. A cost function of linear regression is used to minimize the Root Mean Squared Error (RMSE) between predicted y value and experimental y value (y).

#### Ridge regression

Linear regression with a tuning model is the Ridge regression method. It is used where multi-collinearity is observed. Unbiased least-square and significant variance are the results of multi-collinearity. The second-order linear regularization was used in the Ridge regression^35^. The cost function is shown by:

2 $$\min \left[ {\left( {y - \theta x} \right)^{2} + \lambda \theta^{2} )} \right]$$

#### LASSO regression

The Ridge regressions are replaced with the LASSO regression with first-order linear regularization where some of the variables do not contribute effectively to the prediction task. Then the coefficients of the corresponding variables would be zero. It led to reforming the model function to a selective model and enhanced the prediction task^36^.

#### Elastic Net regression

Where, the ridge and lasso regression are both limited to L1 and L2 norms, the elastic net could be used without these limited conditions ignoring the penalties of ridge and LASSO regressions. Ultimately, the elastic net regression could be used efficiently for linear regression. The elastic net could be applied in complex regression problems with simplicity^37^.

#### Support vector machine (SVM)

The reduction of the elastic net to linear regression with support shows efficient results in the optimization problems. A training dataset with linear SVM learns and separates the data in a classification setting and parameterizes it by a weight vector for a unique solution^38^.

#### Decision tree

IT is a powerful ML method for regression and classification of complex datasets to perform multi-output tasks. Also, the foundation of random forest regression is a more complex method for regression that would be explained. The most critical parameter in this method is the depth or layer of the decision tree. It could be used to perform regression and as a predictor to evaluate the optimized value of the label with the corresponding selected features with more bias and lower variance concerning the other methods^39^.

#### Gradient boosting decision tree regression

This method is considered in this research. It is an ensemble learning method that is coupled with a decision tree. It uses the shrinkage regularization technique. This method also supports a subsample hyper-parameter that specifies the fraction of training increases to train the trees. As could be deducted, this technique is a higher bias for lower variance. The main advantage of this method is the speeded-up training process^40^.

## Data Availability

The datasets generated and/or analyzed during the current study are not publicly available due to [REASON WHY DATA ARE NOT PUBLIC] but are available from the corresponding author on reasonable request.
